# L-asparaginase induces IP3R-mediated ER Ca^2+^ release by targeting µ-OR1 and PAR2 and kills acute lymphoblastic leukemia cells

**DOI:** 10.1038/s41420-024-02142-9

**Published:** 2024-08-15

**Authors:** Jung Kwon Lee, Hamza Kamran, Ki-Young Lee

**Affiliations:** Department of Cell Biology & Anatomy, Arnie Charbonneau Cancer and Alberta Children’s Hospital Research Institutes, University of Calgary, Calgary, AB Canada

**Keywords:** Acute lymphocytic leukaemia, Chemotherapy

## Abstract

L-asparaginase is a standard therapeutic option for acute lymphoblastic leukemia (aLL), a hematologic cancer that claims the most lives of pediatric cancer patients. Previously, we demonstrated that L-asparaginase kills aLL cells via a lethal rise in [Ca^2+^]_i_ due to IP3R-mediated ER Ca^2+^ release followed by calpain-1-Bid-caspase-3/12 activation (Blood, 133, 2222-2232). However, upstream targets of L-asparaginase that trigger IP3R-mediated ER Ca^2+^ release remain elusive. Here, we show that L-asparaginase targets µ-OR1 and PAR2 and induces IP3R-mediated ER Ca^2+^ release in aLL cells. In doing so, µ-OR1 plays a major role while PAR2 plays a minor role. Utilizing PAR2- and µ-OR1-knockdown cells, we demonstrate that L-asparaginase stimulation of µ-OR1 and PAR2 relays its signal via G_αi_ and G_αq_, respectively. In PAR2-knockdown cells, stimulation of adenylate cyclase with forskolin or treatment with 8-CPT-cAMP reduces L-asparaginase-induced µ-OR1-mediated ER Ca^2+^ release, suggesting that activation of µ-OR1 negatively regulates AC and cAMP. In addition, the PKA inhibitor 14-22 amide (myr) alone evokes ER Ca^2+^ release, and subsequent L-asparaginase treatment does not induce further ER Ca^2+^ release, indicating the involvement of PKA inhibition in L-asparaginase-induced µ-OR1-mediated ER Ca^2+^ release, which can bypass the L-asparaginase-µ-OR1-AC-cAMP loop. This coincides with (a) the decreases in PKA-dependent inhibitory PLCβ3 Ser1105 phosphorylation, which prompts PLCβ3 activation and ER Ca^2+^ release, and (b) BAD Ser118 phosphorylation, which leads to caspase activation and apoptosis. Thus, our findings offer new insights into the Ca^2+^-mediated mechanisms behind L-asparaginase-induced aLL cell apoptosis and suggest that PKA may be targeted for therapeutic intervention for aLL.

## Introduction

Acute lymphoblastic leukemia (aLL) is a devastating cancer that mostly affects children. It comprises over a quarter of all pediatric cancers, causing most of the cancer death among children [1]. L-asparaginase, a key component of aLL chemotherapy, is thought to deplete asparagine from plasma, the major asparagine source for cancer cells, resulting in cellular asparagine deficit, inhibition of protein synthesis, and subsequent apoptosis [2]. This established view, however, was confronted by the occurrence of L-asparaginase resistance in some patients. The proposed mechanisms behind resistance include antibody-induced inactivation [3] and overexpression of asparaginase synthetase [4, 5], which synthesizes asparagine from other amino acids [6]. However, the fact that asparagine synthetase level does not always correlate with L-asparaginase resistance [7–9] implies the presence of other mechanisms for L-asparaginase resistance in aLL cells.

By using RNAi screening and knockdown studies, we previously identified huntingtin-associated protein 1 (HAP1) [10] and μ-opioid receptor 1 (µ-OR1; also known as OPRM1 or MOR1) [11] as L-asparaginase resistance biomarkers in aLL cells. Loss of HAP1 or µ-OR1 in aLL cells is linked to L-asparaginase resistance, indicating that L-asparaginase induces aLL cell apoptosis through pathways that involve HAP1 [10] and µ-OR1 [11].

HAP1 acts together with huntingtin (Htt) and the intracellular Ca^2+^ channel, inositol 1,4,5-trisphosphate (IP3) receptor (IP3R), to form a ternary HAP1-Htt-IP3R complex that permits IP3-mediated ER Ca^2+^ release [10]. Thus, absence of HAP1 in aLL cells, which counteracts the complex formation and thus ER Ca^2+^ release, grants resistance to L-asparaginase [10]. In contrast, presence of HAP1 in aLL cells, which promotes the formation of a functional HAP1-Htt-IP3R ternary complex and L-asparaginase-induced ER Ca^2+^ release, triggers perturbation of intracellular Ca^2+^ homeostasis and stimulation of an apoptotic pathway that includes calpain-1, Bid, cyt C, and caspase-3 and -12 [10]. Ca^2+^ chelation by BAPTA-AM [1,2-bis(2-aminophenoxy)ethane-*N,N,N*’,*N*’-tetraacetic acid tetrakis(acetoxymethyl ester)], which reverses the L-asparaginase apoptotic effect in aLL cells, proves a direct tie between L-asparaginase-induced excessive [Ca^2+^]_i_ elevation and apoptotic killing of aLL cells [10]. However, the upstream target(s) of L-asparaginase that triggers HAP1-mediated ER Ca^2+^ release remains obscure.

A clue for the identification of L-asparaginase targets emerged from our investigation on a synthetic opioid, D,L-methadone, that was previously put forth as an anticancer agent, particularly for leukemia [12, 13] and glioblastoma [13, 14]. D,L-methadone was shown to sensitize leukemia [12, 15] and glioblastoma cells [14] to doxorubicin treatment. Although D,L-methadone was reported to stimulate opioid receptor, its specific target in aLL cells and how it kills aLL cells remained unclear. This was partly due to the presence of four different types of opioid receptors: the classical mu (μ), kappa (κ) and delta (δ) opioid receptors, and the nociceptin/orphanin FQ receptor (aka opioid receptor-like receptor) [16] and the fact that the mu opioid receptor consists of three subtypes: mu1, mu2, and morphine-6β-glucuronide (M6G) [17]. Using knockdown studies and single-cell Ca^2+^ imaging, we recently showed that D,L-methadone specifically targets µ-OR1 and induces aLL cell apoptosis by deregulating IP3R-mediated ER Ca^2+^ release and rate of Ca^2+^ extrusion, causing a lethal rise in [Ca^2+^]_i_ that upregulated the calpain-1-Bid-cytochrome C-caspase-3/12 apoptotic pathway [18]. We also demonstrated that chelating intracellular Ca^2+^ with BAPTA-AM rescued aLL cell apoptosis upon D,L-methadone treatment, establishing a direct connection between the lethal rise in [Ca^2+^]_i_ and D,L-methadone-induced aLL cell apoptosis [18]. Since our previous studies [10] indicated that presence and absence of µ-OR1 determine the fate of aLL cells following treatment of L-asparaginase: i.e., presence leads to cell death while absence leads to survival or resistance, µ-OR1 may serve as a L-asparaginase target, killing aLL cells through a mechanism similar to that described above by D,L-methadone. However, this awaits further investigation.

µ-OR1 is a typical G-protein-coupled receptor (GPCR), stimulation of which instigates alteration in receptor conformation, which permits GDP to GTP exchange on G protein α subunit (G_α_), resulting in unpairing of G_α_ from G_βγ_ [19]. Both G_βγ_ dimer and GTP-bound G_α_ relay signaling through their downstream effectors such as Ca^2+^ or cyclic adenosine monophosphate (cAMP). Dissociated G_βγ_ stimulates phospholipase C beta (PLCβ) that hydrolyzes phosphatidylinositol-4,5-bisphosphate (PIP2) into inositol 1,4,5-trisphosphate (IP3) and diacylglycerol. IP3 then triggers endoplasmic reticulum (ER) Ca^2+^ release via IP3 receptor (IP3R) Ca^2+^ channel. Conversely, GTP-bound G_αi_ inhibits adenylyl cyclase (AC) activity, resulting in reduced [cAMP]_i_ [15]. Since cAMP activates protein kinase A (PKA), which phosphorylates (a) PLCβ3 at Ser1105, inhibiting PLCβ3 activation [20], and (b) BAD at Ser118, disrupting the BCL-2-BAD interaction, which stimulates BCL-2 anti-apoptotic activity [21, 22], it is possible that L-asparaginase induces aLL cell apoptosis through downregulation of PKA activity due to ↓[cAMP]_i_ which can cause reduced phosphorylation of (a) PLCβ3 at Ser1105, stimulating PLCβ3 activity and IP3R-mediated ER Ca^2+^ release, and (b) BAD at Ser118, promoting the formation of the BCL2-BAD complex, which neutralizes BCL2 anti-apoptotic activity, allowing BAK and BAX to form pores in the outer mitochondrial membrane, inducing cyt C release, caspase activation, and apoptosis [23, 24].

Another clue arises from the observation that another GPCR, protease-activated receptor 2 (PAR2), is being activated as a side-effect when aLL patients were treated with L-asparaginase [25]. Although this event happens a low frequency, activated PAR2 causes perturbation of Ca^2+^ homeostasis and necrosis, leading to acute pancreatitis [25]. Thus, it is possible that PAR2 may also serve as another cellular target for L-asparaginase in aLL cells. Since PAR2 primarily interacts with G_αq_ and G_αi_ [26], and activates PLC, cleaving phosphatidylinositol 4,5-bisphosphate (PIP2) into diacylglycerol (DAG) and IP3 [27], it will be interesting to determine whether, in aLL cells, L-asparaginase stimulates PAR2 and elicits IP3-IP3R-mediated ER Ca^2+^ release via G_αq_-PLCβ activation, causing an excessive increase in [Ca^2+^]_i_ and ultimately apoptosis.

In this study, we utilized small molecular inhibitors, knockdown strategies and single-cell Ca^2+^ imaging to further characterize the L-asparaginase-induced µ-OR1- and PAR2-signaling cascades for ER Ca^2+^ release, leading to apoptosis. We demonstrate that L-asparaginase stimulates µ-OR1-G_αi_-AC-↓[cAMP]_i_-↓PKA-↓pSer1105-PLCβ3 and PAR2-G_αq_-PLCβ3 pathways to induce IP3R-mediated ER Ca^2+^ release in aLL cells.

## Results

### L-asparaginase triggers ER Ca^2+^ release in aLL cells via stimulation of µ-OR1 and PAR2

**Fig. 1:**
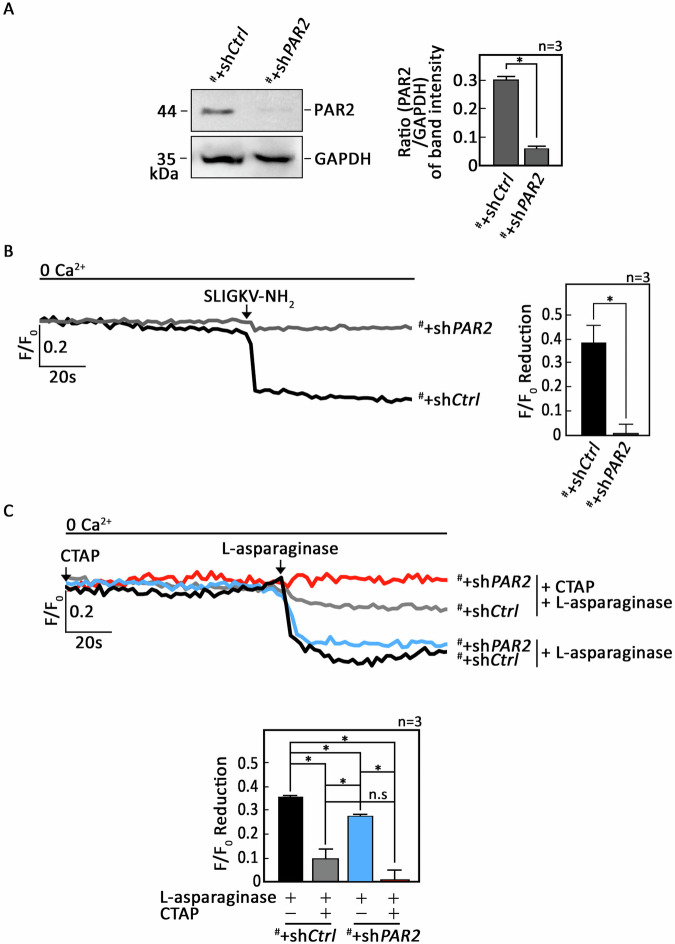
L-asparaginase evokes ER Ca^2+^ release in aLL cells through stimulation of µ-OR1 and PAR2. **A** Lysates of SEM cells (^#^) infected with lentivirus carrying control (^#^+sh*Ctrl*) or PAR2 shRNA (^#^+sh*PAR2*) were resolved by SDS-PAGE and immunoblotted for PAR2. GAPDH blot was used as loading control. The right panel shows the ratios of PAR2 vs GAPDH levels measured by densitometric analysis of the blots using NIH ImageJ 1.61. GAPDH levels were normalized to 1.0. Standard deviations were calculated from three sets of experiments (*n* = 3). **B** ^#^+sh*Ctrl* and ^#^+sh*PAR2* cells loaded with Mag-Fluo-4 AM were stimulated with SLIGKV-NH_2_ and analyzed for ER Ca^2+^ release using single-cell Ca^2+^ imaging. (C) ^#^+sh*Ctrl* and ^#^+sh*PAR2* cells loaded with Mag-Fluo-4 AM were pretreated (or not pretreated) with CTAP then treated with L-asparaginase and analyzed for ER Ca^2+^ release. Left panel in **B** and upper panel in C show the average Ca^2+^ tracings taken every second from 10 individual cells before and after treatments. Data are from one of three independent experiments (*n* = 3) showing similar results. Charts on the right **B** and lower **C** panels show the difference in ER Ca^2+^ release following treatment with L-asparaginase pretreated (or not pretreated) with CTAP. An F/F_0_ value of 30 sec after L-asparaginase addition (left panel in **B** and upper panel in **C**) was used to determine F/F_0_ reduction. Values are means ± SEM from the three independent experiments. *p < 0.05, N.S., not significant.

To investigate whether L-asparaginase-induced IP3R-mediated ER Ca^2+^ release in aLL cells [10] results from stimulation of µ-OR1 and/or PAR2, SEM cells (^#^) originating from the peripheral blood of a relapsed 5-year-old female B-aLL patient [10] were infected with lentivirus carrying sh*PAR2* (^#^+sh*PAR2*) to knock down PAR2 (Fig. 1A/Supplemental Material) and tested whether they lost their ability to induce PAR2-mediated ER Ca^2+^ release. For this experiment, cells were loaded with an ER Ca^2+^ probe, Mag-Fluo-4 AM [28], then treated with a synthetic PAR2 agonist peptide, SLIGKV-NH_2_, which mimics the tethered human PAR2 ligand [29]. By single-cell Ca^2+^ imaging, we found that ^#^+sh*PAR2* cells failed to exhibit PAR2-mediated ER Ca^2+^ release (Fig. 1B). Next, cells loaded with Mag-Fluo-4 AM then treated (or untreated) with CTAP, a µ-OR1 specific inhibitor [30], to block µ-OR1-mediated ER Ca^2+^ release, were exposed to L-asparaginase and analyzed for ER Ca^2+^ release. As shown in Fig. 1C, treatment with L-asparaginase alone elicited ER Ca^2+^ release in both ^#^+sh*Control* (^#^+sh*Ctrl*) and ^#^+sh*PAR2* cells, but to a lesser extent (24 ± 3%; lower panel, bars 1 vs 3) in ^#^+sh*PAR2* cells, indicating the involvement of PAR2 in L-asparaginase-induced ER Ca^2+^ release. Pre-treatment of ^#^+sh*Ctrl* cells with CTAP drastically reduced the extent of ER Ca^2+^ release (72 ± 9%; lower panel, bars 1 vs 2) following L-asparaginase treatment, indicating a major role for µ-OR1 in L-asparaginase-induced release of Ca^2+^ from the ER. The fact that pretreatment of ^#^+sh*PAR2* cells with CTAP completely blocked ER Ca^2+^ release (lower panel, bars 3 vs 4 & bars 1 vs 4) indicates that L-asparaginase evokes ER Ca^2+^ release in aLL cells through two independent receptor pathways: µ-OR1 and PAR2, with µ-OR1 playing a major role while PAR2 playing a minor role (24 ± 3% to 28 ± 9%; Fig. 1C, lower panel, bar 3 in ^#^+sh*PAR2* cells to bar 2 in ^#^+sh*Ctrl* cells, respectively). Next, we tested whether this observation can be recapitulated in a different aLL cells. To do so, POETIC2 cells (^P^) originating from a 14-year-old patient with pre-B aLL [11] were infected with lentivirus carrying sh*PAR2* (^P^+sh*PAR2*) to knock down PAR2 (Supplementary Figure 1A/Supplemental Material). These cells then treated (or untreated) with CTAP were exposed to L-asparaginase and analyzed for ER Ca^2+^ release. As shown in Supplementary Figure 1B and 1C, similar pattern of ER Ca^2+^ release was also occurred in POETIC2 aLL cells.

**Fig. 2:**
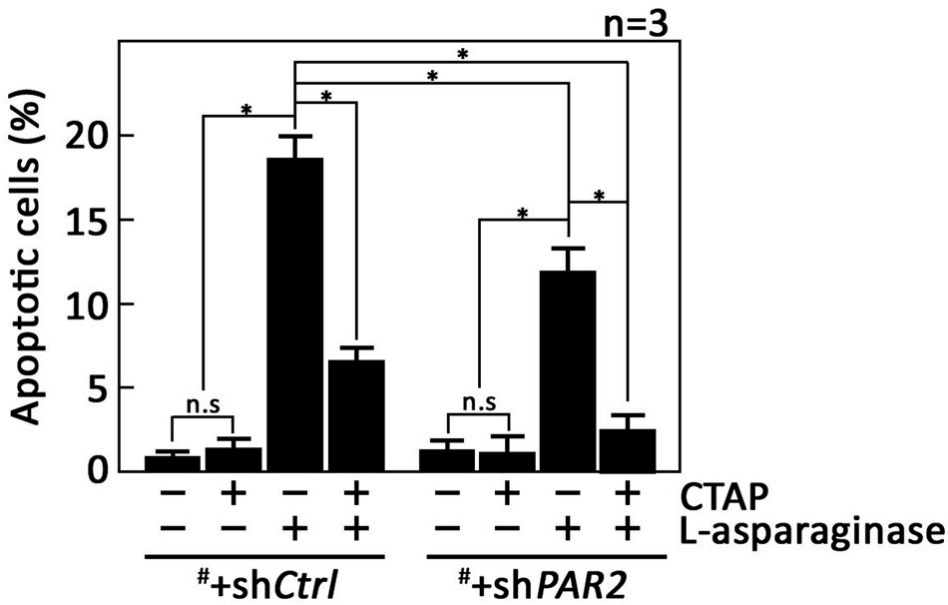
L-asparaginase induces aLL cell apoptosis via stimulation of µ-OR1 and PAR2. ^#^+sh*Ctrl* and ^#^+sh*PAR2* cells pretreated (or not pretreated) with CTAP for 3 h then treated L-asparaginase for 16 h were double-stained with PI and FITC-labeled Annexin V. FITC-positive apoptotic cells were counted at 10× magnification using a I×71 Olympus inverted microscope attached to a 37 °C incubator with 5% CO_2_. The percentage of FITC-positive apoptotic cells was determined from a field of 80 to 120 PI-stained cells using the Olympus CellSens software (Olympus, Japan). *p < 0.05, N.S., not significant.

Next, we sought to examine the extent of aLL cell apoptosis upon stimulation of µ-OR1 or PAR2 by L-asparaginase. To do so, ^#^+sh*Ctrl* and ^#^+sh*PAR2* cells pretreated (or not pretreated) with CTAP then treated with L-asparaginase were double-stained with propidium iodide (PI) and FITC-labeled Annexin V. FITC-positive apoptotic cells were counted 16 h post-treatment and the percentage of apoptotic cells was determined. As shown in Fig. 2, pretreatment of CTAP inhibited L-asparaginase-induced apoptosis by 66 ± 3% (bar 4) in ^#^+sh*Ctrl* cells and almost 90% (bar 8) in ^#^+sh*PAR2* cells. L-asparaginase treatment in cells depleted of PAR2 (^#^+sh*PAR2* cells) resulted in a considerably lesser extent (65 ± 8%; bar 1 vs 7) of apoptotic cell death. These observations are consistent with our data indicating that L-asparaginase elicits ER Ca^2+^ release through stimulation of µ-OR1 and PAR2 and that µ-OR1 plays a greater role in L-asparaginase-induced ER Ca^2+^ release than PAR2. Similar pattern of aLL cell apoptosis was also observed in POETIC2 cells (Supplementary Fig. 2).

### L-asparaginase stimulation of µ-OR1 and PAR2 relays signaling through G_αi_ and G_αq_, respectively

**Fig. 3:**
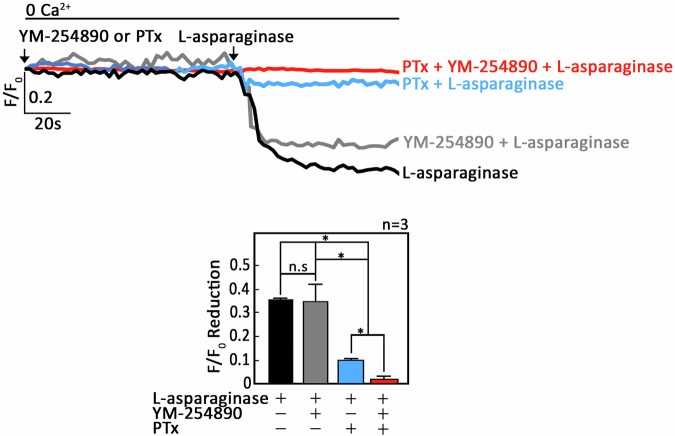
L-asparaginase causes ER Ca^2+^ release in aLL cells through µ-OR1-G_αi_ and PAR2-G_αq_ pathways. **A** ^#^+sh*Ctrl* cells loaded with Mag-Fluo-4 AM then pretreated (or not pretreated) with PTx or YM-254890, or both, were exposed to L-asparaginase and analyzed for ER Ca^2+^ release by single-cell Ca^2+^ imaging. The top panel shows the average Ca^2+^ tracings per sec from 10 individual cells before and after treatments. Data are from one of three independent experiments (*n* = 3) showing similar results. The chart on the bottom shows the difference in ER Ca^2+^ release following treatment with L-asparaginase pretreated (or not pretreated) with YM-254890 or PTx. An F/F_0_ value of 40 s after L-asparaginase addition (left panel) was used to determine F/F_0_ reduction. Values are means ± SEM of the three independent experiments. *p < 0.05, N.S., not significant.

**Fig. 4:**
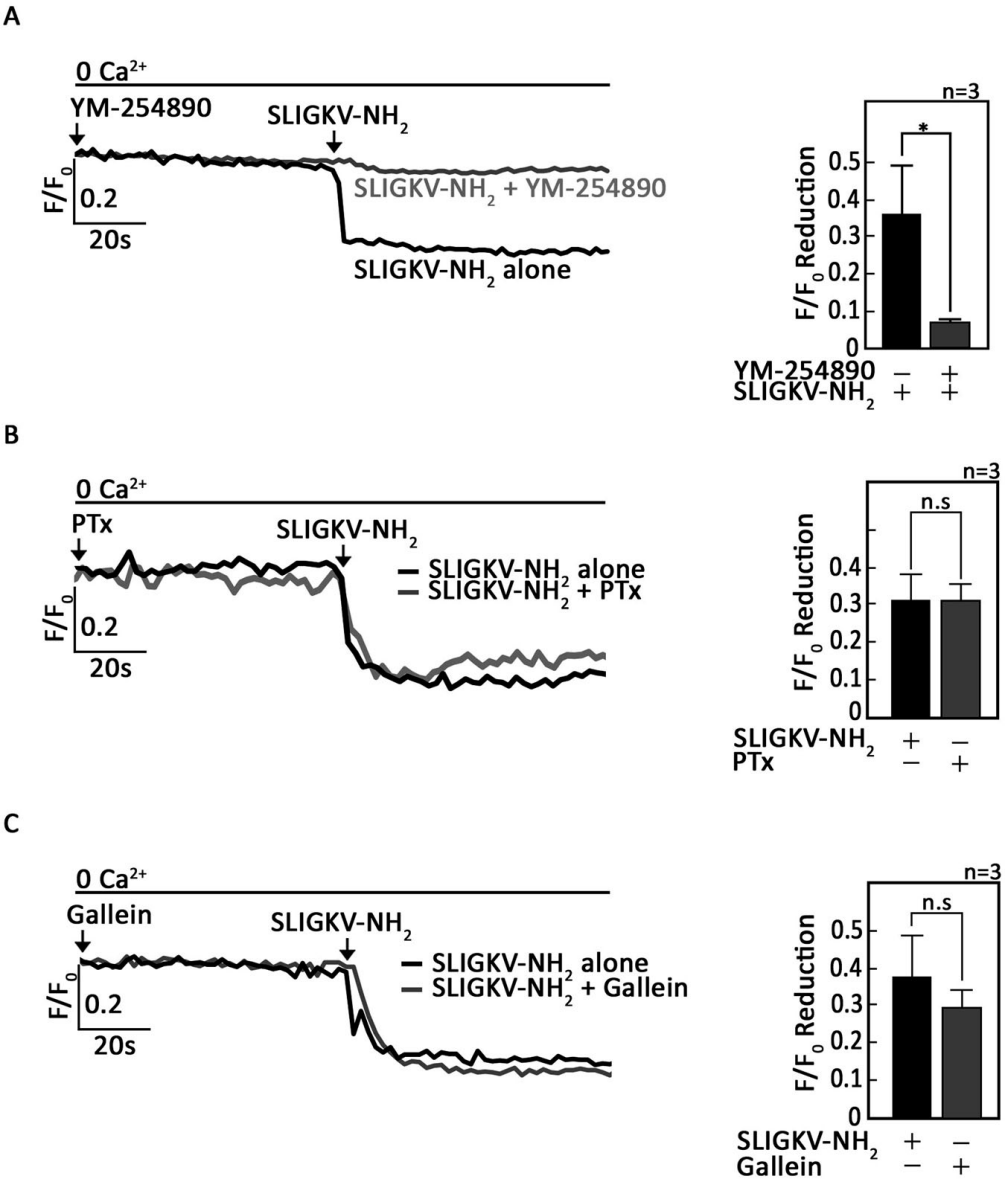
Stimulation of PAR2 using SLIGKV-NH_2_ reveals signaling through G_αq_. **A** ^#^+sh*Ctrl* cells loaded with Mag-Fluo-4 AM were pre-treated with YM-254890 then exposed to SLIGKV-NH_2_ and analyzed for ER Ca^2+^ release by single-cell Ca^2+^ imaging. **B**, **C** ^#^+sh*Ctrl* cells loaded with Mag-Fluo-4 AM then pretreated (or not pretreated) with PTx **B** or Gallein **C** were exposed to the SLIGKV-NH2 and analyzed for ER Ca^2+^ release. The left panels show the average Ca^2+^ tracing taken every second from 10 individual cells before and after treatments. Data are from one of three independent experiments (*n* = 3) showing similar results. The charts on the right show the difference in ER Ca^2+^ release following treatment with SLIGKV-NH_2_ pretreated (or not pretreated) with YM-254890, PTx or Gallein. An F/F_0_ value of 40 s after SLIGKV-NH_2_ addition (left panel) was used to determine F/F_0_ reduction. Values are means ± SEM from the three independent experiments. *p < 0.05, N.S., not significant.

To determine whether signaling through G_αi_ and G_αq_ holds true for L-asparaginase stimulation of µ-OR1 and PAR2, respectively, ^#^+sh*Ctrl* cells loaded with Mag-Fluo-4-AM then pretreated (or not pretreated) with a G_αi_ inhibitor, PTx [31], and/or a G_αq_ inhibitor, YM-254890 [32], were exposed to L-asparaginase and analyzed for ER Ca^2+^ release. As shown in Fig. 3, PTx inhibited L-asparaginase-induced ER Ca^2+^ release in ^#^+sh*Ctrl* cells by up to 72 ± 5% (lower panel, bar 3). While YM-254890 also inhibited ER Ca^2+^ release, it did not reach significance (lower panel, bar 2). Treatment of ^#^+sh*Ctrl* cells with both PTx and YM-254890 completely blocked ER Ca^2+^ release (lower panel, bar 4). As signaling through G_αq_ during L-asparaginase-induced PAR2-mediated ER Ca^2+^ release was inconclusive due to the fact that PAR2-mediated ER Ca^2+^ release was not robust upon L-asparaginase stimulation, we further explored the involvement of G_αq_ in PAR2-mediated ER Ca^2+^ release. To do so, ^#^+sh*Ctrl* cells loaded with Mag-Fluo-4-AM then pretreated (or not pretreated) with YM-254890 then exposed to SLIGKV-NH_2_, and analyzed for ER Ca^2+^ release. Pretreatment with YM-254890 almost completely blocked PAR2-mediated ER Ca^2+^ release (Fig. 4A), suggesting that, like SLIGKV-NH_2_, L-asparaginase may induce PAR2-mediated ER Ca^2+^ release through G_αq_. We also checked the potential absence of G_αi_ and G_βϒ_ involvement in PAR2-mediated ER Ca^2+^ release. For this experiment, ^#^+sh*Ctrl* cells loaded with Mag-Fluo-4-AM then pretreated (or not pretreated) with PTx or a potent G_βϒ_ inhibitor, Gallein [33], were exposed to SLIGKV-NH_2_, and analyzed for ER Ca^2+^ release. As shown in Figs. 4B and 4C, pre-treatment with PTx (4B) or Gallein (4 C) had no effect in PAR2-mediated ER Ca^2+^ release, suggesting that, like SLIGKV-NH_2_, L-asparaginase may induce PAR2-mediated ER Ca^2+^ release via G_αq_ but not G_βϒ_ and G_αi_.

**Fig. 5:**
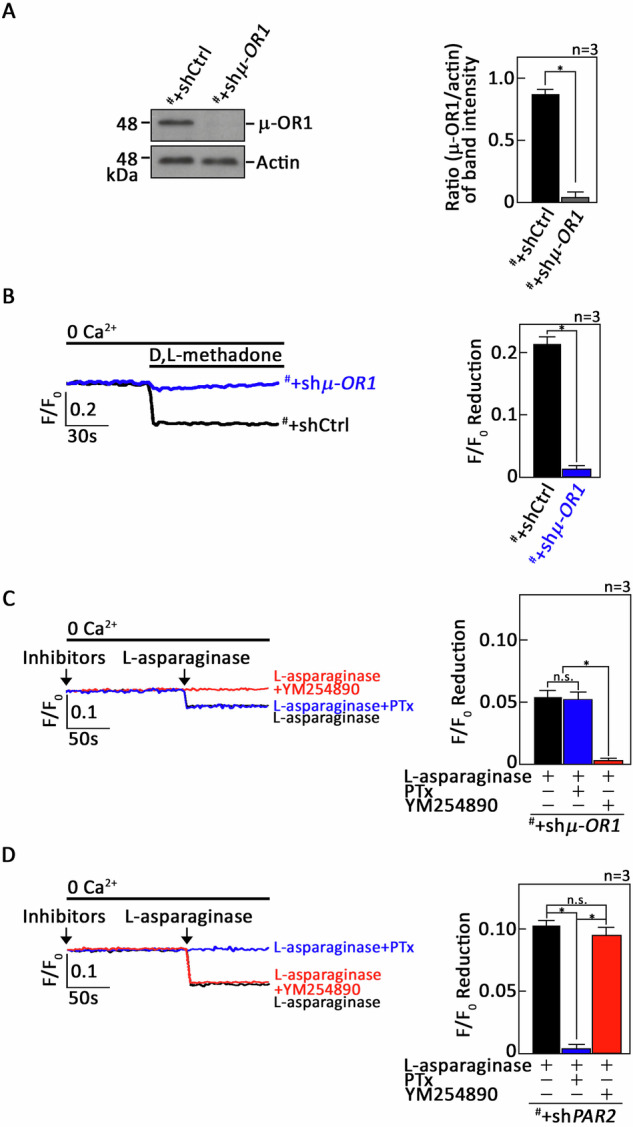
L-asparaginase-induced ER Ca^2+^ release via µ-OR1 and PAR2 are mediated by G_αi_ and G_αq_, respectively. **A** Lysates of SEM cells (^#^) infected with lentivirus carrying control (^#^+sh*Ctrl*) or sh*µ-OR1* shRNA (^#^+sh*µ-OR1*) were resolved by SDS-PAGE and immunoblotted for µ-OR1. Actin blot was used as loading control. The right panel shows the ratios of µ-OR1 vs actin levels measured by densitometric analysis of the blots using NIH ImageJ 1.61. Actin levels were normalized to 1.0. Standard deviations were calculated from three sets of experiments (*n* = 3). **B** ^#^+sh*Ctrl* and ^#^+sh*µ-OR1* cells loaded with Mag-Fluo-4 AM were stimulated with D,L-methadone and analyzed for ER Ca^2+^ release using single-cell Ca^2+^ imaging. (C and D) ^#^+sh*µ-OR1* **C** and ^#^+sh*PAR2* **D** cells loaded with Mag-Fluo-4 AM were pretreated (or not pretreated) with YM254890 or PTx then treated with L-asparaginase and analyzed for ER Ca^2+^ release using single-cell Ca^2+^ imaging. The left panels show the Ca^2+^ tracings taken every second from 15 individual cells before and after treatments. Data are from one of three independent experiments (*n* = 3) showing similar results. The charts on the right show the difference in ER Ca^2+^ release following treatment with L-asparaginase pretreated (or not pretreated) with YM-254890 or PTx. An F/F_0_ value of 50 s after L-asparaginase addition (left panel) was used to determine F/F_0_ reduction. Values are means ± SEM from the three independent experiments. *p < 0.05, N.S., not significant.

To validate our suggestion that L-asparaginase-induced ER Ca^2+^ release via µ-OR1 and PAR2 are mediated by G_αi_ and G_αq_, respectively, SEM cells (^#^) were infected with lentivirus carrying sh*µ-OR1* (^#^+sh*µ-OR1*) to knock down µ-OR1 (Fig. 5A/Supplemental Material) and tested first whether they lost their ability to evoke µ-OR1-mediated ER Ca^2+^ release. To do so, cells were loaded with Mag-Fluo-4 AM [28], then treated with a synthetic opioid, D,L-methadone [18] and analyzed for ER Ca^2+^ release. As shown in Fig. 5B, we found that ^#^+sh*µ-OR1* cells failed to exhibit µ-OR1-mediated ER Ca^2+^ release. Next, ^#^+sh*µ-OR1* and ^#^+sh*PAR2* cells loaded with Mag-Fluo-4 AM then treated (or untreated) with YM-254890 and PTx were exposed to L-asparaginase and analyzed for ER Ca^2+^ release. As shown in Figs. 5C and 5D, YM-254890 completely inhibited L-asparaginase-induced ER Ca^2+^ release in ^#^+sh*µ-OR1* cells (Fig. 5C), while PTx completely inhibited L-asparaginase-induced ER Ca^2+^ release in ^#^+sh*PAR2* cells (Fig. 5D). ^#^+sh*µ-OR1* and ^#^+sh*PAR2* cells pretreated with PTx and YM-254890, respectively, were used as negative controls. Taken together, our findings indicate that L-asparaginase-induced ER Ca^2+^ release via µ-OR1 and PAR2 is mediated via G_αi_ and G_αq_, respectively. Same pattern of YM-254890 and PTx inhibition of L-asparaginase-induced ER Ca^2+^ release in ^P^+sh*µ-OR1* cells (Supplementary Figure 5A) and ^P^+sh*PAR2* cells (Supplementary Figure 5B), respectively, was also detected in POETIC2 aLL cells.

### Adenylate cyclase (AC), cAMP and PKA are regulated negatively during L-asparaginase-induced µ-OR1-mediated ER Ca^2+^ release

**Fig. 6:**
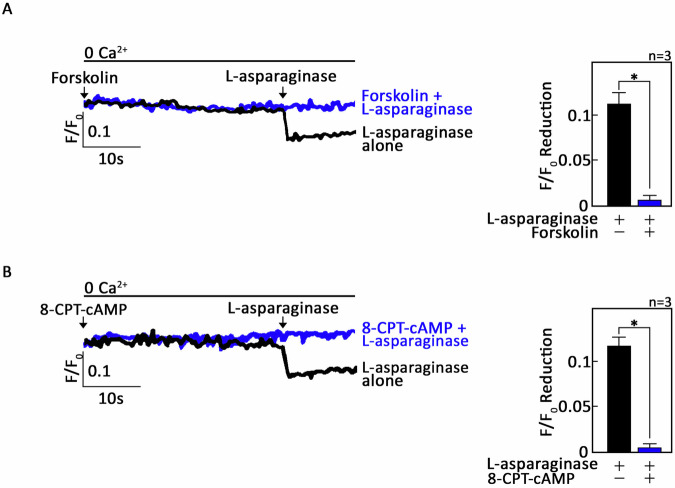
Stimulation of AC with forskolin or treatment with exogenous 8-CPT-cAMP in PAR2-knockdown aLL cells blocks L-asparaginase-induced ER Ca^2+^ release. ^#^+sh*PAR2* cells loaded with Mag-Fluo-4 AM were subjected to Ca^2+^ tracing via single-cell Ca^2+^ imaging. After obtaining stable baseline ER Ca^2+^ levels, the cells were pretreated (or not pretreated) with forskolin **A** or 8-CPT-cAMP **B** and then treated with L-asparaginase to analyze ER Ca^2+^ release. Left panels show the average Ca^2+^ tracing measured per second in 10 individual cells after forskolin **A** or 8-CPT-cAMP **B** treatment. Data are from one of three independent experiments (*n* = 3) showing similar results. Charts on the right show the difference in ER Ca^2+^ release following treatment with L-asparaginase pretreated (or not pretreated) with forskolin **A** or 8-CPT-cAMP **B**. An F/F_0_ value of 5 s after L-asparaginase addition (left panel) was used to determine F/F_0_ reduction. Values are means ± SEMs from the three independent experiments. *p < 0.05.

Next, we determined if G_αi_-dependent ER Ca^2+^ release, which is mediated by µ-OR1 upon L-asparaginase treatment, involves AC and cAMP. To do so, ^#^+sh*PAR2* and ^P^+sh*PAR2* cells loaded with Mag-Fluo-4 AM were pretreated with forskolin, an AC activator [34], or 8-CPT-cAMP, an exogenous cell permeable cAMP analog. These cells were then treated with L-asparaginase and assessed for ER Ca^2+^ release. As shown in Fig. 6 and Supplementary Figure 4, forskolin and 8-CPT-cAMP abolished L-asparaginase-induced ER Ca^2+^ release, indicating that AC and cAMP are implicated in this process.

**Fig. 7:**
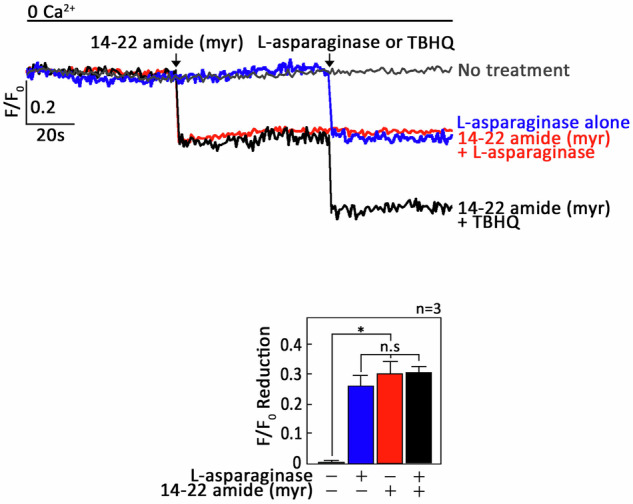
Treatment with 14-22 amide (myr) alone elicits ER Ca^2+^ release in in PAR2-knockdown aLL cells cells, and subsequent treatment with L-asparaginase does not cause additional ER Ca^2+^ release. ^#^+sh*PAR2* cells loaded with Mag-Fluo-4 AM were subjected to Ca^2+^ tracing via single-cell Ca^2+^ imaging. After obtaining stable baseline ER Ca^2+^ levels, cells were pretreated (or not pretreated) with 14–22 amide (myr) for 60 s then treated with L-asparaginase or TBHQ for 40 s to analyze ER Ca^2+^ release. Upper panel shows the average Ca^2+^ tracing measured per second in 10 individual cells after 14–22 amide (myr) treatment. Treatment with TBHQ, an ER Ca^2+^ pump inhibitor, caused further ER Ca^2+^ release, indicating that these cells were viable during analysis. The data are from one of three independent experiments (*n* = 3) showing similar results. Chart on the bottom shows the difference in ER Ca^2+^ release following treatment with L-asparaginase pretreated (or not pretreated) with 14–22 amide (myr). An F/F_0_ value of 20 s after D,L-methadone and/or 14–22 amide (myr) addition (left panel) was used to determine the F/F_0_ reduction. The values are presented as the means ± SEMs of three independent experiments. *p < 0.05. N.S., not significant.

Because cAMP controls [Ca^2+^]_i_ by the inhibitory phosphorylation of PLCβ3 through PKA [20, 35, 36], it is conceivable that µ-OR1 activation by L-asparaginase, which induces ER Ca^2+^ release, may also involve PKA inhibition. If this holds true, we anticipate that treatment with 14–22 amide (myr), a myristylated cell-permeable PKA-specific inhibitor [37], alone will induce ER Ca^2+^ release. In addition, pretreatment with 14–22 amide (myr) will not evoke a further increase in L-asparaginase-induced ER Ca^2+^ release. As shown in Fig. 7 and Supplementary Fig. 5, 14–22 amide (myr) alone elicited ER Ca^2+^ release (Ca^2+^ tracings in red and blue, respectively), and following treatment with L-asparaginase did not cause further ER Ca^2+^ release (Ca^2+^ tracings in red and blue, respectively). These findings indicate that µ-OR1-G_αi_-AC-cAMP-dependent L-asparaginase-induced ER Ca^2+^ release involves PKA inhibition, which can bypass the upstream L-asparaginase-µ-OR1-AC-cAMP step. Treatment with TBHQ, an ER Ca^2+^ pump inhibitor, caused further ER Ca^2+^ release, indicating that these cells were viable during analysis (Fig. 7 and Supplementary Fig. 5).

### L-asparaginase-induced µ-OR1-mediated ER Ca^2+^ release is associated with the downregulation of PKA-mediated (a) PLCβ3 inhibitory phosphorylation at Ser1105 and (b) BAD phosphorylation at Ser118

**Fig. 8:**
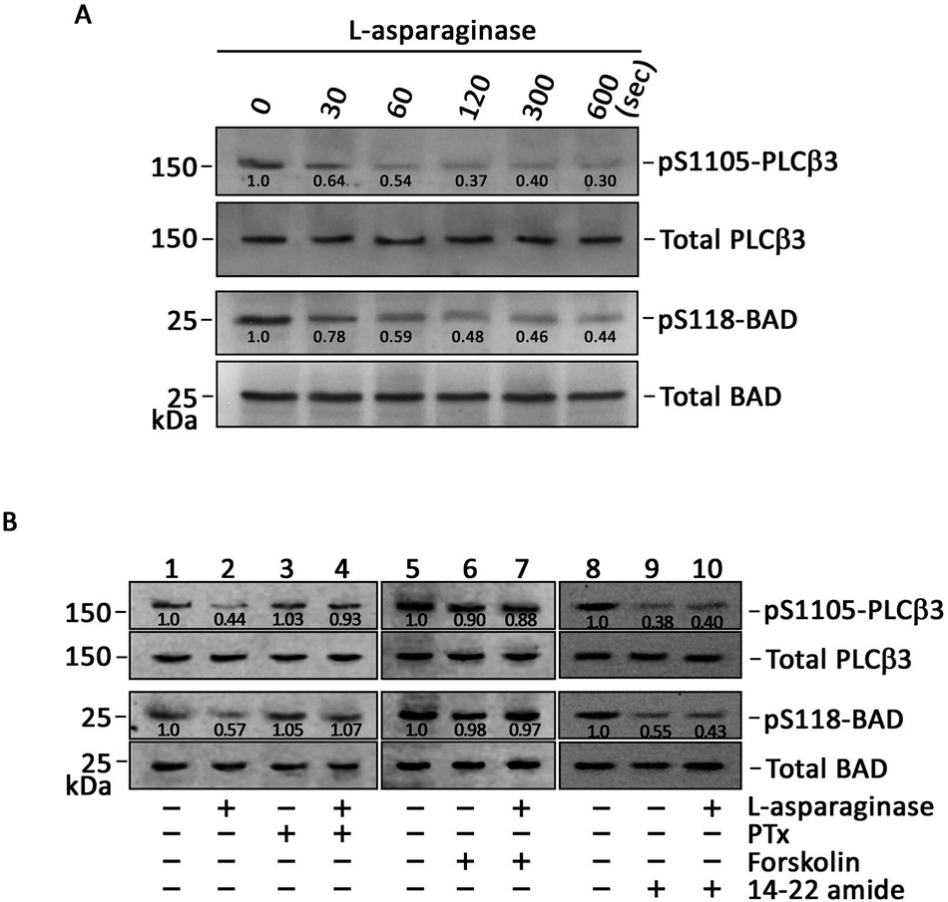
L-asparaginase-induced ER Ca^2+^ release in aLL cells is associated with the downregulation of PLCβ3 at Ser1105 and BAD at Ser118 phosphorylations. Lysates of ^#^+sh*PAR2* cells pretreated (or not pretreated: **A** with PTx (**B**, lanes 3 and 4), forskolin (B, lanes 6 and 7) or 14–22 amide (myr; lanes 9 and 10) then stimulated with L-asparaginase for 120 s were subjected to SDS‒PAGE and immunoblotting for pSer1105-PLCβ3 and total PLCβ3, and pSer118-BAD and total BAD. Numbers under pSer1105-PLCβ3 and pSer118-BAD bands represent relative intensity ratios of the pSer1105-PLCβ3 or pSer118-BAD vs total PLCβ3 or BAD bands, respectively, with values at time 0 normalized to 1.

**Fig. 9:**
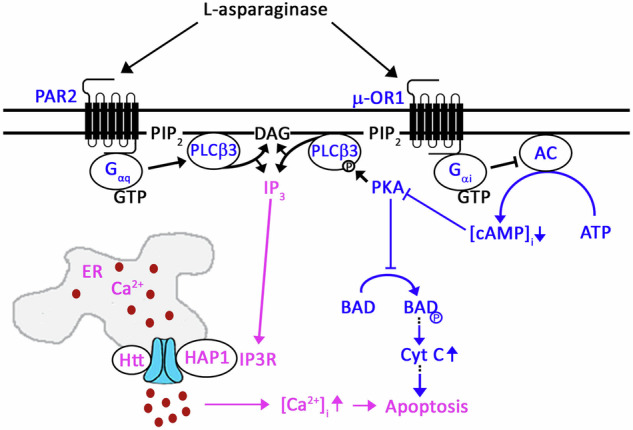
Proposed pathways for L-asparaginase-induced IP3R-mediated ER Ca^2+^ release and apoptosis in aLL cells. We previously showed that L-asparaginase induces aLL cell apoptosis via an excessive rise in [Ca^2+^]_i_ due to IP3R-mediated ER Ca^2+^ release followed by calpain-1-Bid-caspase-3/12 activation (in pink) [10]. In the current study, we demonstrate that L-asparaginase triggers IP3R-mediated ER Ca^2+^ release in aLL cells via two independent receptor pathways: µ-OR1-G_αi_-↓AC-↓[cAMP]_i_-↓PKA-↓pSer1105-PLCβ and PAR2-G_αq_ (in blue). The former is also linked by a decrease in PKA-mediated ↓pSer118-BAD (in blue), stimulating apoptotic pathway in aLL cells.

Next, we tested if L-asparaginase negatively regulates PKA-mediated inhibitory phosphorylation of PLCβ3 at Ser1105, which is required for PLC-IP3/IP3R-mediated ER Ca^2+^ release [38]. To do so, lysates of ^#^+sh*PAR2* cells treated with L-asparaginase for indicated time periods were resolved by SDS-PAGE and subsequently immunoblotted for pSer1105-PLCβ3. As shown in Fig. 8A/Supplemental Material, L-asparaginase clearly decreased the level of pSer1105-PLCβ3, which concurred with the ER Ca^2+^ release upon L-asparaginase treatment, as shown in Fig. 7 (Ca^2+^ tracing in blue). As expected, pretreatment with PTX or forskolin did not affect L-asparaginase-induced PLCβ3 phosphorylation at Ser1105 (Fig. 8B/Supplemental Material), which is consistent with the loss of L-asparaginase-induced ER Ca^2+^ release upon PTX or forskolin treatment, respectively (Figs. 5D and 6A, respectively). As cAMP activates PKA, which phosphorylates BAD at Ser118, disrupting the BCL2-BAD interaction, which stimulates BCL2 antiapoptotic activity [21, 22], we tested if L-asparaginase also negatively regulates BAD phosphorylation at Ser118. Figure 8A/Supplemental Material shows that, as with pSer1105-PLCβ3, the level of pSer118-BAD was decreased upon L-asparaginase treatment. Pretreatment with PTX or forskolin did not affect L-asparaginase-induced BAD phosphorylation at Ser118 (Fig. 8B/Supplemental Material). To determine the consequence of PKA inhibition in PLCβ3 activation, lysates of ^#^+sh*PAR2* cells pretreated with 14-22 amide (myr) then treated with L-asparaginase were subjected to SDS‒PAGE and immunoblotting for pSer1105-PLCβ3 and pSer118-BAD. As shown in Fig. 8B (right panel)/Supplemental Material, treatment with L-asparaginase or 14-22 amide (myr) noticeably decreased the phosphorylation of PLCβ3 at Ser1105 and BAD at Ser118, indicating that such phosphorylation occurs through PKA. Our observation that 14-22 amide (myr) alone caused a substantial decrease in PLCβ3 phosphorylation at Ser1105 is consistent with our finding that 14-22 amide (myr) alone evoked ER Ca^2+^ release and suggests that L-asparaginase-induced µ-OR1-mediated ER Ca^2+^ release occurs via the G_αi_-AC-cAMP-PKA-PLCβ3 pathway (Fig. 9). Similar pattern of decreased levels of pSer1105-PLCβ3 and pSer118-BAD were also observed in POETIC2 cells (Supplementary Figure 6/Supplemental Material).

## Discussion

L-asparaginase has been a key component of aLL chemotherapy regimens since the late 1960s [2]. The established mode of action for L-asparaginase: i.e., the depletion of asparagine from plasma and subsequent inhibition of protein synthesis [2] was recently challenged by a breakthrough finding emerged from unbiased genome-wide RNAi screening and knockdown studies [10, 11]: i.e., perturbation of [Ca^2+^]_i_ homeostasis. With this advancement in our understanding, it is imperative that we define how L-asparaginase kills aLL cells in order to gain an insight into potential targets for the therapeutic interventions in aLL.

As summarized in Fig. 9, we demonstrate that L-asparaginase, which induced IP3R-mediated ER Ca^2+^ release and subsequently aLL cell apoptosis [10], targets two transmembrane GPCRs, µ-OR1 and PAR2, which possess an extracellular N-terminal region, seven transmembrane domains, and an intracellular C-terminal tail. The former was identified as a L-asparaginase resistance biomarker [11]; the absence of which causes survival while presence of which kills aLL cells. A clue for the identification of the latter as a L-asparaginase target arises from an unexpected but intriguing observation that approximately 5-10% of aLL patients who were treated with L-asparaginase suffered from acute pancreatitis as a side-effect and that L-asparaginase caused PAR2-mediated rise in [Ca^2+^]_i_ in pancreatic acinar cells, which was associated with acute pancreatitis [25]. Certainly, our data showing significantly reduced levels of L-asparaginase-induced ER Ca^2+^ release in aLL cells pretreated with µ-OR1-specific inhibitor, CTAP [30] (72 ± 9%), and lacking PAR2 (24 ± 3% to 28 ± 9%), respectively, establish that L-asparaginase targets µ-OR1 and PAR2. The notion that L-asparaginase induces ER Ca^2+^ release only via two independent receptors, µ-OR1 and PAR2, was corroborated by our data showing that pretreatment of aLL cells depleted of PAR2 with CTAP completely obliterates ER Ca^2+^ release. In terms of causing the extent of L-asparaginase-induced ER Ca^2+^ release, we note that µ-OR1 plays a greater role while PAR2 participates in a lesser degree, which was further supported by our finding that the similar extent of aLL cell apoptosis occurred upon stimulation of µ-OR1 and/or PAR2 by L-asparaginase. Whether or not L-asparaginase targets asparagine residue(s) of µ-OR1- and PAR2 via its amidohydrolase (EC 3.5.1.1) activity [39] or β-aspartyl peptidase activity [39–42], and whether other protein(s) are involved in the process awaits further investigation.

Since stimulation of GPCRs such as µ-OR1 and PAR2 alters their conformation that promotes GDP to GTP exchange in specific subsets of G-proteins to relay signals, we used the G-protein selective inhibitors as well as PAR2- and µ-OR1-knockdown aLL cells to demonstrate that L-asparaginase-induced ER Ca^2+^ release via µ-OR1 and PAR2 are mediated by G_αi_ and G_αq_, respectively. These findings are in line with previously published works showing that G_αi_ serves as a downstream effector for µ-OR1 stimulation in aLL cells [15], and G_αq_ for PAR2 in HEK293 and COS7 cells [29, 43]. It is also not surprising that the extent (~72 ± 5%) by which PTx, a G_αi_ inhibitor, inhibited L-asparaginase-induced ER Ca^2+^ release in ^#^+sh*Ctrl* cells was matched with the extent of inhibition by CTAP (72 ± 9%) in ^#^+sh*Ctrl* cells, indicating that L-asparaginase induces ER Ca^2+^ release predominantly through the µ-OR1-G_αi_ pathway.

Since G_αi_-mediated inhibition of AC downregulates [cAMP]_i_ which in turn inhibits PKA activity, we investigated the involvement of AC and cAMP in the µ-OR1-G_αi_ cascade in PAR2-knockdown cells. Our data showing that activation of AC with forskolin and treatment with exogenous 8-CPT-cAMP abolished L-asparaginase-induced ER Ca^2+^ release substantiates our finding that µ-OR1-mediated ER Ca^2+^ release is dependent on G_αi_ and that L-asparaginase downregulates [cAMP]_i_ by inducing G_αi_-mediated inhibition of AC activity. In addition, our finding that the 14-22 amide (myr) alone caused ER Ca^2+^ release, and that subsequent L-asparaginase addition did not cause further ER Ca^2+^ release indicates that inhibition of PKA activity is involved in L-asparaginase-induced µ-OR1-mediated ER Ca^2+^ release.

Next, we tested if PLCβ3 and BAD serve as downstream effectors of the µ-OR1-G_αi_-AC-cAMP-PKA cascade. Our data revealed that L-asparaginase instigated PKA-mediated downregulation of PLCβ3 phosphorylation at Ser1105, and that pretreatment with PTx or forskolin did not influence pSer1105-PLCβ3 level upon L-asparaginase-treatment. We also demonstrated that L-asparaginase reduced pSer118-BAD level. These findings are consistent with the previous reports showing that downregulated PKA activates PLCβ3, causing IP3/IP3R-mediated ER Ca^2+^ release [20] and that reduced pSer118-BAD level due to downregulated PKA activity stimulates the BCL2-BAD complex formation, which nullifies the BCL2 anti-apoptotic activity, permitting BAK and BAX to produce pores in the outer mitochondrial membrane, prompting the release of cyt C, caspase cleavage, and apoptosis [23, 24].

Our analysis of the GSE134759 dataset from the NCBI Gene Expression Omnibus (GEO) database indicated that the expression levels of PAR2 and µ-OR1, in aLL patients, were considerably lower at the time of relapse compared to the time of diagnosis: 1.45 ± 0.07 vs 3.2 ± 0.14; *p* = 0.004 for PAR2 and 1.10 ± 0.14 vs 2.65 ± 0.21; *p* ≤ 0.05 for µ-OR1, respectively. These data link PAR2 and µ-OR1 levels with relapse, and suggests that PAR2 and µ-OR1 levels could be used to predict aLL patient treatment outcome. However, absence of data sets in available databases prevented us from further acquiring a relationship between PAR2 and µ-OR1 levels and L-asparaginase resistance.

While aLL patients who lack µ-OR1 are resistance to L-aspagainase [11], our finding that inhibition of PKA activity by the 14–22 amide (myr) can bypass the upstream µ-OR1-AC-cAMP step implies that PKA may be targeted for therapy in L-asparaginase-refractory aLL.

## Material and methods

### Materials

RPMI 1640, penicillin-streptomycin, fetal bovine serum (FBS), Mag-Fluo-4 AM and antibodies against µ-OR1 (PA5-26138) and phosphorylated Ser118 BAD (PA5-12550) were from ThermoFisher Scientific (Burlington, ON, Canada). D,L-methadone was from Market Drugs Medical Ltd (Edmonton, AB). *E. coli* L-asparaginase (L-asparaginase II; ab277068), SLIGKV-NH_2_ (ab141809), CTAP (ab120680) and 8-CPT-cAMP (ab120424) were from Abcam (Toronto, ON). Pertussis toxin (PTx), polybrene, annexin V-FITC/PI, 14-22 amide (myr) and 2,5-Di- tert-butylhydroquinone (TBHQ, 112976) were from Sigma-Aldrich (Oakville, ON, Canada). YM-254890 (29735) was from Cayman Chemical (Michigan, USA). Forskolin (66575-29-9) was from Alomone Labs (Jerusalem, Israel). Gallein (sc-202631), antibodies for total PLCβ3 (D-7), PAR2 (SAM11), GAPDH (0411) and BAD (C-7), and lentiviral particles carrying sh*PAR2* (sc-36188-V) or sh*µ-OR1* (sc-35957-V) were from Santa Cruz Biotechnology (Dallas, TX, USA). Antibody against phosphorylated Ser1105 PLCβ3 (2484) was from Cell Signalling (Whitby, ON, Canada).

### Cell culture

SEM [10] and POETIC2 [11] aLL established cell lines were cultured in RPMI1640 and Opti-MEM reduced serum, respectively, at 37 °C and CO_2_ level of 5%. The media were supplemented with 10% fetal bovine serum, and 100 μg/ml penicillin/streptomycin. SEM and POETIC2 cells stably transduced with lentivirus carrying sh*PAR2*, sh*µ-OR1* or sh*Control* (sh*Ctrl*) shRNA were generated according to the manufacturer’s instructions. Briefly, SEM cells grown to 50–60% confluence in 6-well plates were infected with lentivirus carrying control or *PAR2* shRNA in the presence of 5 µg/ml polybrene followed by four consecutive passages of 10 μg/ml puromycin-selected cells. Cells were tested for mycoplasma contamination.

### Measurement of endoplasmic reticulum (ER) Ca^2+^ release

SEM and POETIC2 ( ~ 0.5 × 10^6^) grown on 0.2 mg/ml poly-L-ornithine-coated 12 mm glass coverslips were loaded with 2 μM Mag-Fluo-4 AM in RPMI media for 45 min. Coverslips were then transferred to a 3.5 cm glass bottom plate containing 1 ml of Ca^2+^-free Krebs-Ringer-Henseleit (KRH) buffer (25 mM HEPES, pH 7.4, 125 mM NaCl, 5 mM KCl, 6 mM glucose, 1.2 mM MgCl_2,_ and 2 μM EGTA). Ca^2+^ transients were traced using the DMi8-Film microscope at a magnification of 20× and the LASX imaging software (Leica Microsystems). The HyD laser for confocal imaging (Leica Microsystems) was used at λ_Ex_ = 495 _nm_ and λ_Em_ = 530 _nm_. After obtaining stable baseline ER Ca^2+^ levels, cells were pretreated (or not pretreated) with 10 µM CTAP, 2 µM YM-254890, 0.1 µM PTx, 2 µM Gallein or 2 µM 14-22 amide (myr), 1 μM 8-CPT-cAMP, or 4 μM forskolin for the indicated period of time, then treated with 0.6 µM L-asparaginase, 1 µM SLIGKV-NH_2_, or 1.8 µM D,L-methadone (and subsequently with 10 μM TBHQ). The resulting ER Ca^2+^ release was measured through changes in ER fluorescence: the signal-to-baseline ratio (SBR), which is simply the F/F_0_ ratio, where F= fluorescence value after stimulation and F_0_= basal or initial fluorescence.

### Western blot analysis

Lysates of cells (1 × 10^6^) pretreated (or not pretreated) with 0.1 µM PTx, 4 μM forskolin or 2 µM 14-22 amide (myr) then treated with 0.6 µM L-asparaginase for the indicated period of time were resolved by SDS-PAGE and subjected to immunoblotting for PAR2, µ-OR1, pSer1105-PLCβ3, total PLCβ3, pSer118-BAD, and total BAD. Immunoreactive bands were detected by enhanced chemiluminescence and visualized using the Bio-Rad ChemiDoc Imager at the optimal exposure set up. Ratios of pSer1105-PLCβ3 vs total-PLCβ3 was determined using the NIH ImageJ 1.61 software.

### Apoptotic analysis

^#^+sh*Ctrl* and ^#^+sh*PAR2* cells (1 × 10^4^) seeded in 96-well plates coated with 0.02% poly-L-ornithine were pretreated with 10 µM CTAP for 30 min then treated with 0.6 µM L-asparaginase for 16 h. Cells were double-stained with propidum iodide (PI) and FITC-labeled Annexin V, and visualized at λ_ex_ = 485 nm and λ_em_ = 530 nm and 10× magnification using an IX71 Olympus inverted microscope (Tokyo, Japan). The percentage of FITC-positive apoptotic cells was determined from a field of 80 to 120 PI-stained cells per treatment and analyzed using the Olympus CellSens software (Olympus, Japan). For flow cytometry, cells (0.5 × 10^6^) pretreated with 10 µM CTAP for 3 h then treated with 0.6 µM L-asparaginase for 14 h were harvested, washed twice with 1× PBS, stained with Annexin V-FITC (2 μl) and propidium iodide (2 μl), and analyzed using an Attune NxT flow cytometer (ThermoFisher Scientific, USA).

### Statistical analysis

The student’s unpaired, two-tailed t-test was performed at *p* < 0.05. For experiments that exceeded more than two groups or treatments, one-way Analysis of Variance (ANOVA) with Tukey Honestly Significantly Different (HSD) post hoc tests were conducted to uncover the statistical differences between groups or treatments.

### Supplementary information

Figure legend and supplementary figures 1-6

Uncropped western blots

## Data Availability

All data generated or analyzed during this study are included in this published article and its supplementary information files.
