# Supplementary material for: L-asparaginase induces IP3R-mediated ER Ca2+ release by targeting µ-OR1 and PAR2 and kills acute lymphoblastic leukemia cells
Source: Cell Death Discov. 2024 Aug 15;10:366. doi: 10.1038/s41420-024-02142-9 (PMC11327372; doi:10.1038/s41420-024-02142-9)
Supplement: Supplementary file 1 — Figure legend and supplementary figures 1-6 [file 41420_2024_2142_MOESM1_ESM.pdf]

Supplementary Figure Legend

Supplementary Figure 1. L-asparaginase also induces ER Ca^{2+} release via stimulation of µ-OR1 and PAR2 in POETIC2 aLL cells. (A) Lysates of POETIC2 cells (^{P}) infected with lentivirus carrying control (^{P}+sh*Ctrl*) or PAR2 shRNA (^{P}+sh*PAR2*) were resolved by SDS-PAGE and immunoblotted for PAR2. Actin blot was used as loading control. The right panel shows the ratios of PAR2 vs actin levels measured by densitometric analysis of the blots using NIH ImageJ 1.61. Actin levels were normalized to 1.0. Standard deviations were calculated from three sets of experiments (n=3). (B) ^{P}+sh*Ctrl* and ^{P}+sh*PAR2* cells loaded with Mag-Fluo-4 AM were stimulated with SLIGKV-NH_2 and analyzed for ER Ca^{2+} release using single-cell Ca^{2+} imaging. (C) ^{P}+sh*Ctrl* and ^{P}+sh*PAR2* cells loaded with Mag-Fluo-4 AM then pretreated (or not pretreated) with CTAP were stimulated with L-asparaginase and subsequently analyzed for ER Ca^{2+} release. The upper panel shows the average Ca^{2+} tracings taken every second from 15 individual cells before and after treatments. Data are from one of three independent experiments (n=3) showing similar results. The chart on the bottom show the difference in ER Ca^{2+} release following treatment with L-asparaginase pretreated (or not pretreated) with CTAP. An F/F_0 value of 60 sec after L-asparaginase addition was used to determine F/F_0 reduction. Values are means ± SEM from the three independent experiments. *p<0.05.

Supplementary Figure 2. L-asparaginase also induces POETIC2 aLL cell apoptosis via stimulation of µ-OR1 and PAR2. ^{P}+sh*Ctrl* and ^{P}+sh*PAR2* cells pretreated (or not pretreated) with CTAP for 3 hrs then treated with L-asparaginase for 14 hrs were double-stained with PI and FITC-labeled Annexin V, and subjected to flow cytometry. Values are means ± SEM from three independent experiments (n=3). *p<0.05. N.S., not significant.

Supplementary Figure 3. L-asparaginase-induced ER Ca^{2+} release via µ-OR1 and PAR2 in POETIC 2 aLL cells are also mediated by $G_{\alpha i}$ and $G_{\alpha q}$, respectively. ^{P}+sh*µ-OR1* (A) and ^{P}+sh*PAR2* (B) cells loaded with Mag-Fluo-4 AM were pretreated (or not pretreated) with YM254890 or PTx then treated with L-asparaginase and analyzed for ER Ca^{2+} release using single-cell Ca^{2+} imaging. The left panels show the Ca^{2+} tracings taken every second from 15 individual cells before and after treatments. Data are from one of three independent experiments (n=3) showing similar results. The charts on the right show the difference in ER Ca^{2+} release following treatment with L-asparaginase pretreated (or not pretreated) with YM-254890 or PTx. An F/F_0 value of 50 sec after L-asparaginase addition (left panel) was used to

determine F/F_0 reduction. Values are means ± SEM from the three independent experiments. *$p<0.05$, N.S., not significant.

Supplementary Figure 4. Inhibition of L-asparaginase-induced ER Ca^{2+} release in PAR2-knockdown aLL cells via stimulation of AC with forskolin or treatment with exogenous 8-CPT-cAMP. ^{P}+sh*PAR2* cells loaded with Mag-Fluo-4 AM were subjected to Ca^{2+} tracing via single-cell Ca^{2+} imaging. After obtaining stable baseline ER Ca^{2+} levels, the cells were pretreated (or not pretreated) with forskolin (A) or 8-CPT-cAMP (B) for the indicated time periods then treated with L-asparaginase to analyze ER Ca^{2+} release. Left panels show the average Ca^{2+} tracing measured per second in 15 individual cells after forskolin (A) or 8-CPT-cAMP (B) treatment. Data are from one of three independent experiments (n=3) showing similar results. Charts on the right show the difference in ER Ca^{2+} release following treatment with L-asparaginase pretreated (or not pretreated) with forskolin (A) or 8-CPT-cAMP (B). An F/F_0 value of 40 sec after L-asparaginase addition (left panel) was used to determine F/F_0 reduction. Values are means±SEMs from the three independent experiments. *$p<0.05$.

Supplementary Figure 5. 14-22 amide (myr) causes ER Ca^{2+} release in PAR2-knockdown aLL cells, and subsequent treatment with L-asparaginase does not cause additional ER Ca^{2+} release. ^{P}+sh*PAR2* cells loaded with Mag-Fluo-4 AM were subjected to Ca^{2+} tracing via single-cell Ca^{2+} imaging. After obtaining stable baseline ER Ca^{2+} levels, the cells were pretreated (or not pretreated) with 14-22 amide (myr) for 60 sec then treated with L-asparaginase or TBHQ for 40 sec to analyze ER Ca^{2+} release. Upper panel shows the average Ca^{2+} tracing measured per second in 15 individual cells after 14-22 amide (myr) treatment. Treatment with TBHQ, an ER Ca^{2+} pump inhibitor, caused further ER Ca^{2+} release, indicating that these cells were viable during analysis. The data are from one of three independent experiments (n=3) showing similar results. Chart on the bottom shows the difference in ER Ca^{2+} release following treatment with L-asparaginase pretreated (or not pretreated) with 14-22 amide (myr). An F/F_0 value of 20 sec after D,L-methadone and/or 14-22 amide (myr) addition (left panel) was used to determine the F/F_0 reduction. Values are means±SEMs of three independent experiments. *$p<0.05$. N.S., not significant.

Supplementary Figure 6. L-asparaginase-induced ER Ca^{2+} release in POETIC 2 aLL cells is also associated with the downregulation of PLCβ3 at Ser1105 and BAD at Ser118 phosphorylations. Lysates

of P+sh*PAR2* cells pretreated (or not pretreated: A) with PTx (B, lanes 3 and 4), forskolin (B, lanes 5 and 6) or 14-22 amide (myr; lanes 7 and 8) then stimulated with L-asparaginase for 40 sec were subjected to SDS–PAGE and immunoblotting for pSer1105-PLCβ3 and total PLCβ3, and pSer118-BAD and total BAD. Numbers under pSer1105-PLCβ3 and pSer118-BAD bands represent relative intensity ratios of the pSer1105-PLCβ3 or pSer118-BAD vs total PLCβ3 or BAD bands, respectively, with values at time 0 normalized to 1.

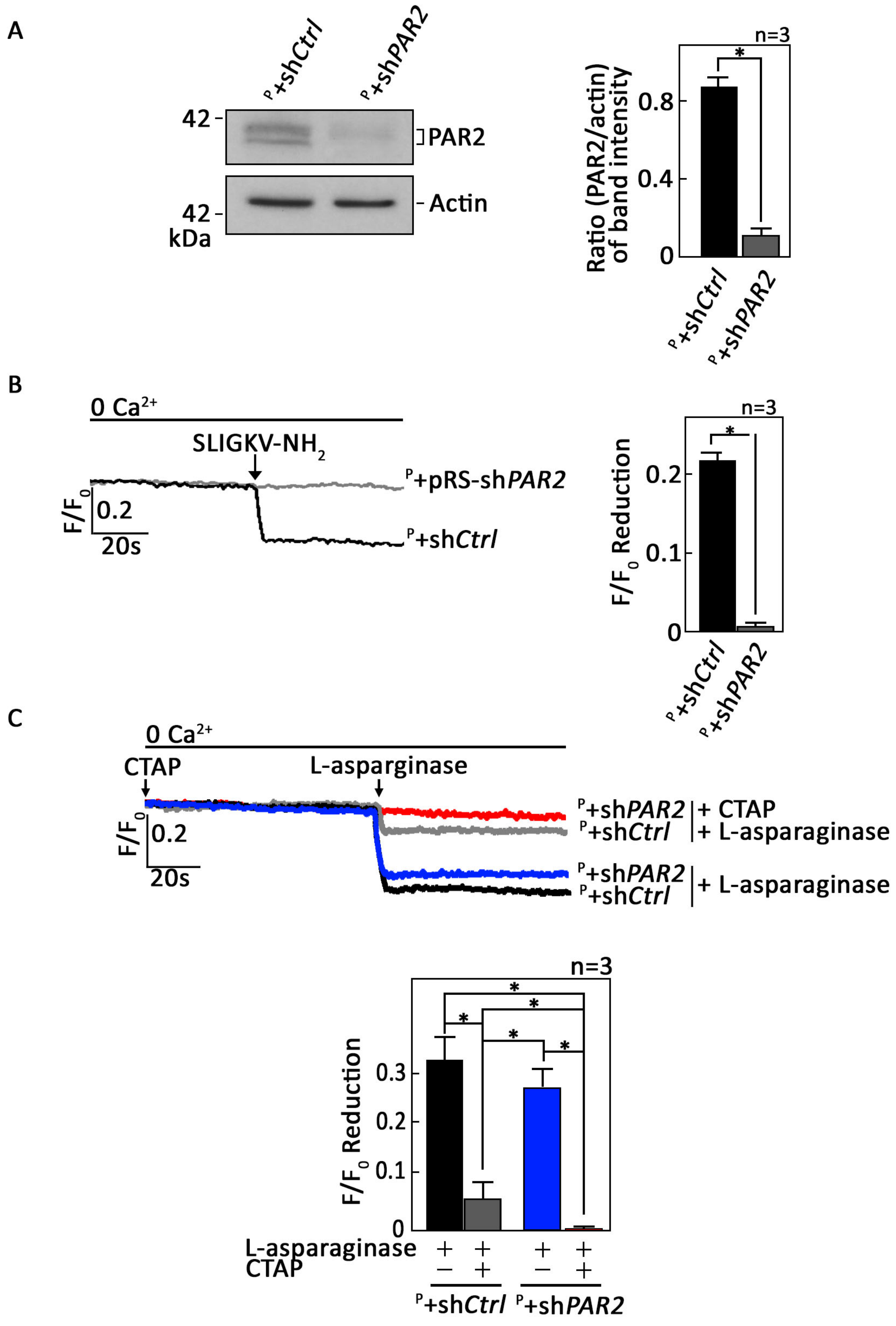

Supplementary Fig.1

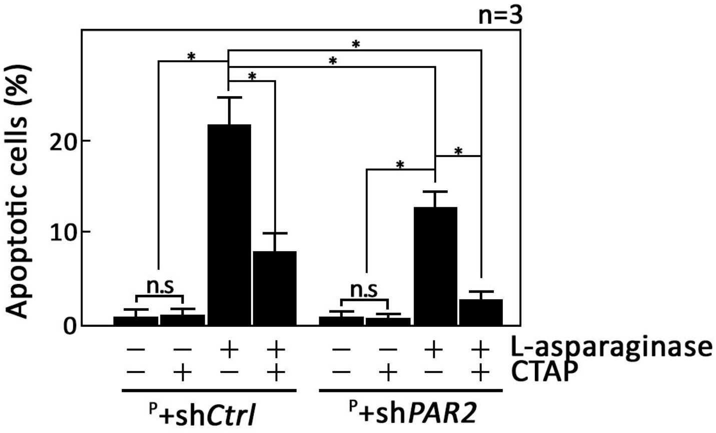

Supplementary Fig. 2

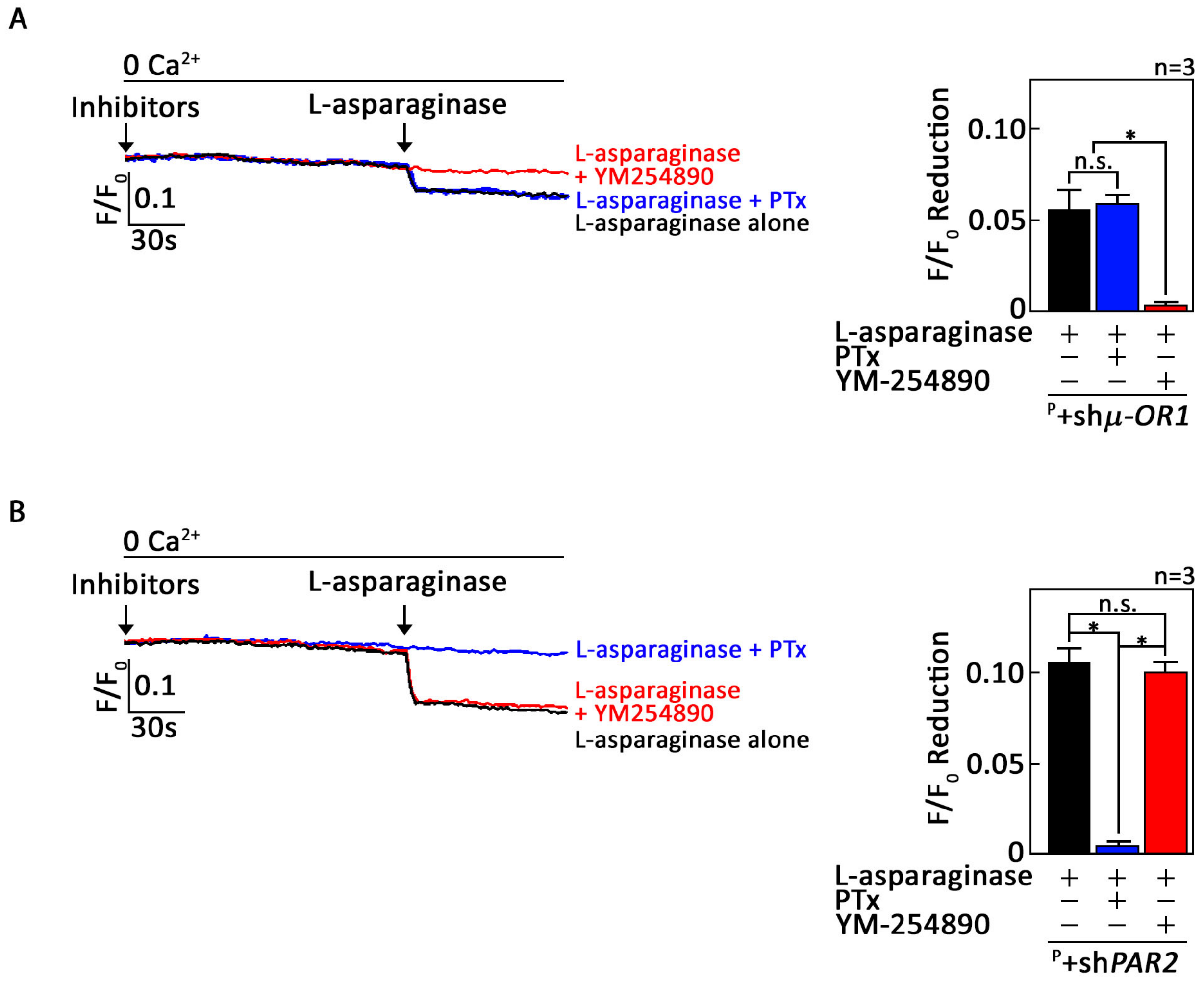

Supplementary Fig. 3

A

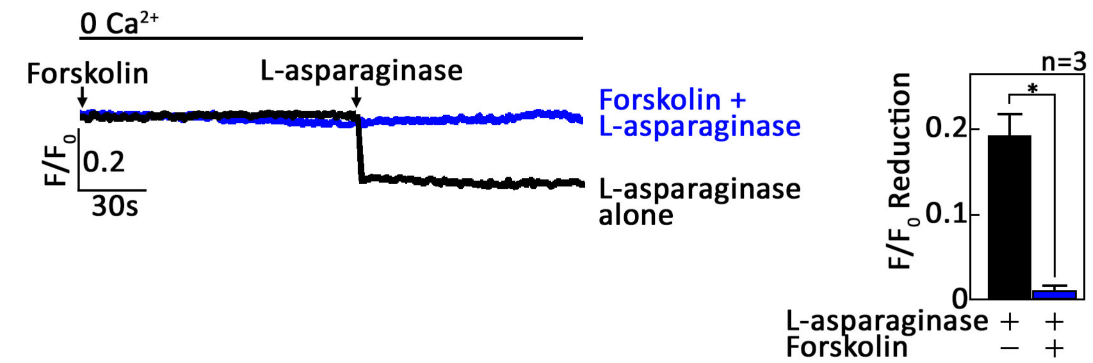

B

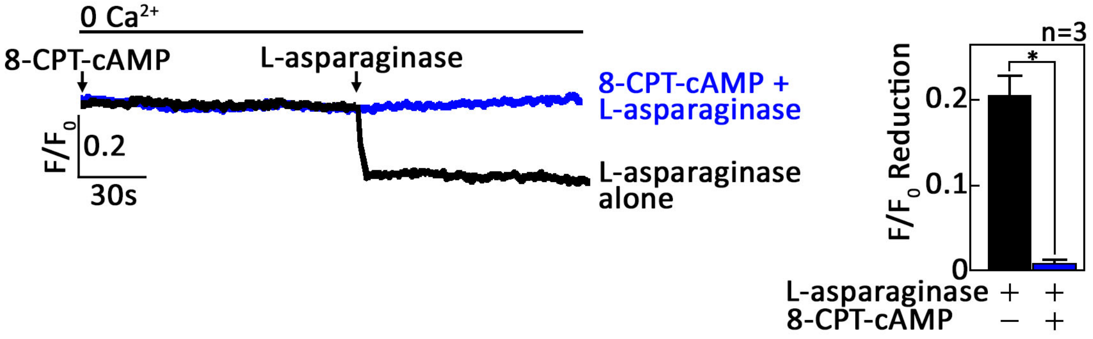

Supplementary Fig. 4

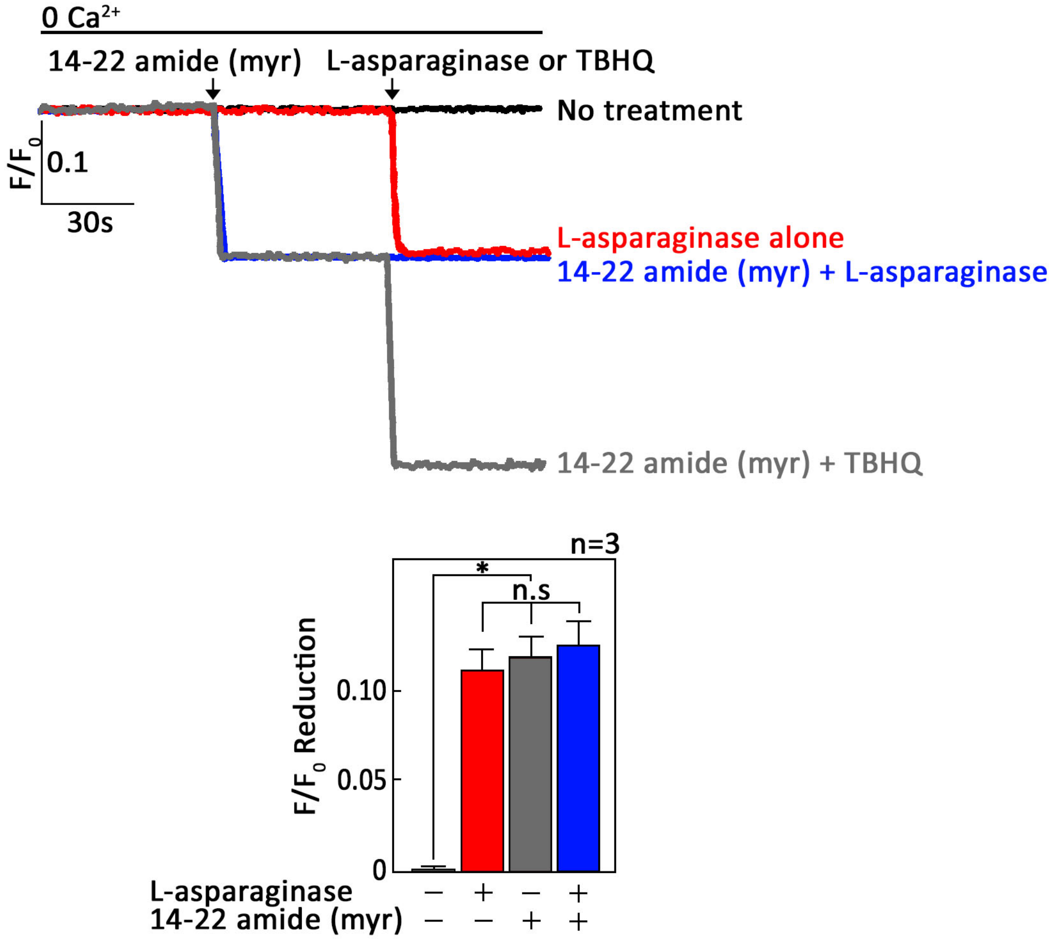

Supplementary Fig. 5

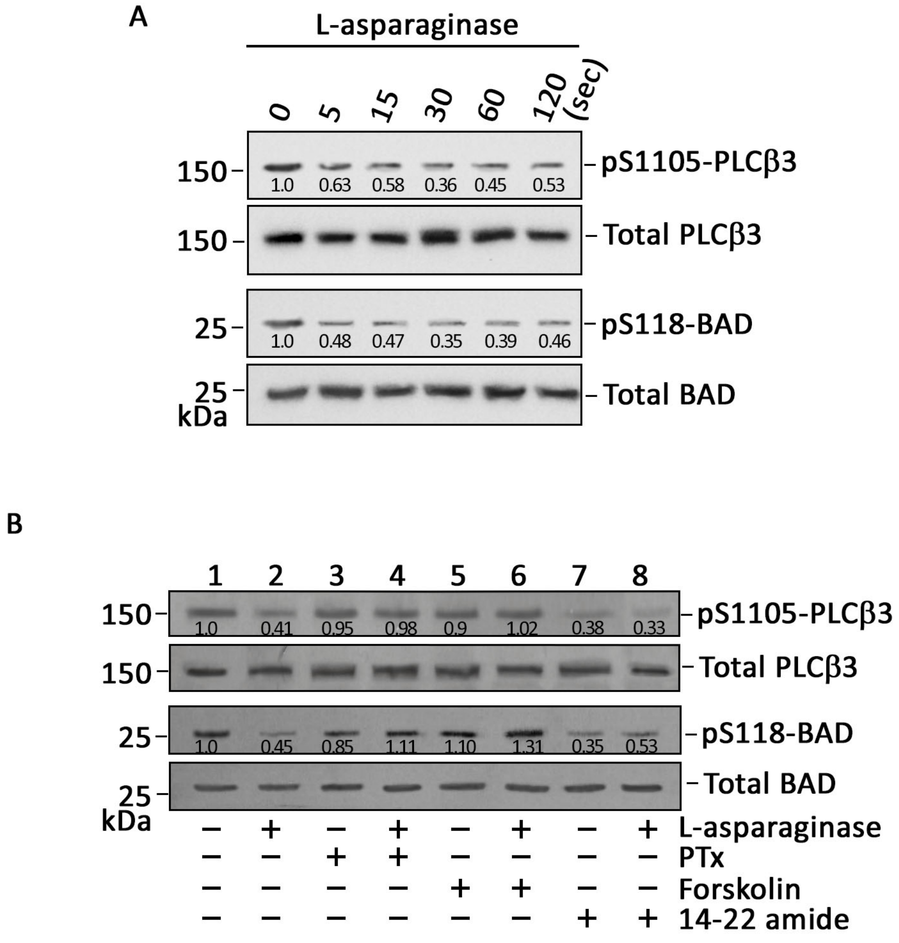

Supplementary Fig. 6
