# Supplementary material for: L-asparaginase induces IP3R-mediated ER Ca2+ release by targeting µ-OR1 and PAR2 and kills acute lymphoblastic leukemia cells
Source: Cell Death Discov. 2024 Aug 15;10:366. doi: 10.1038/s41420-024-02142-9 (PMC11327372; doi:10.1038/s41420-024-02142-9)
Supplement: Supplementary file 2 — Uncropped western blots [file 41420_2024_2142_MOESM2_ESM.pdf]

Figure 1.

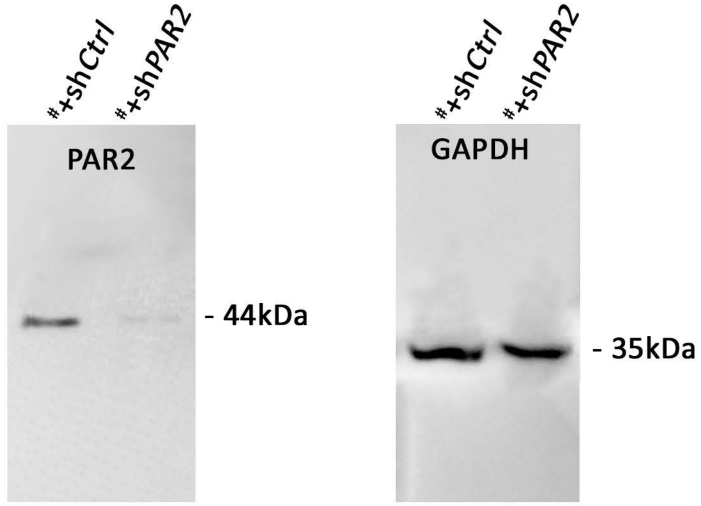

Figure 5.

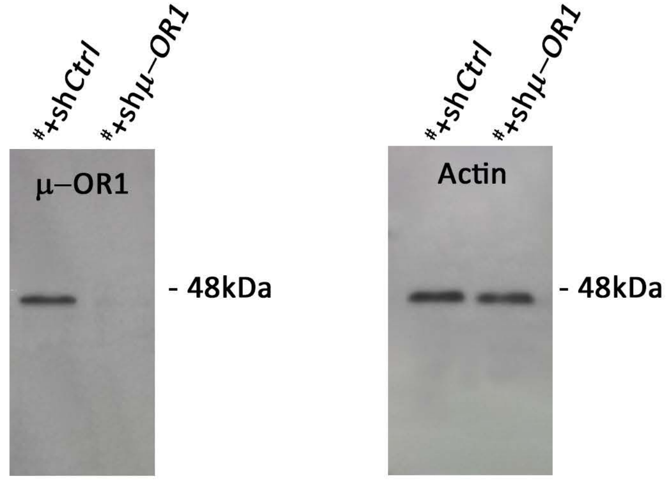

Figure 8A

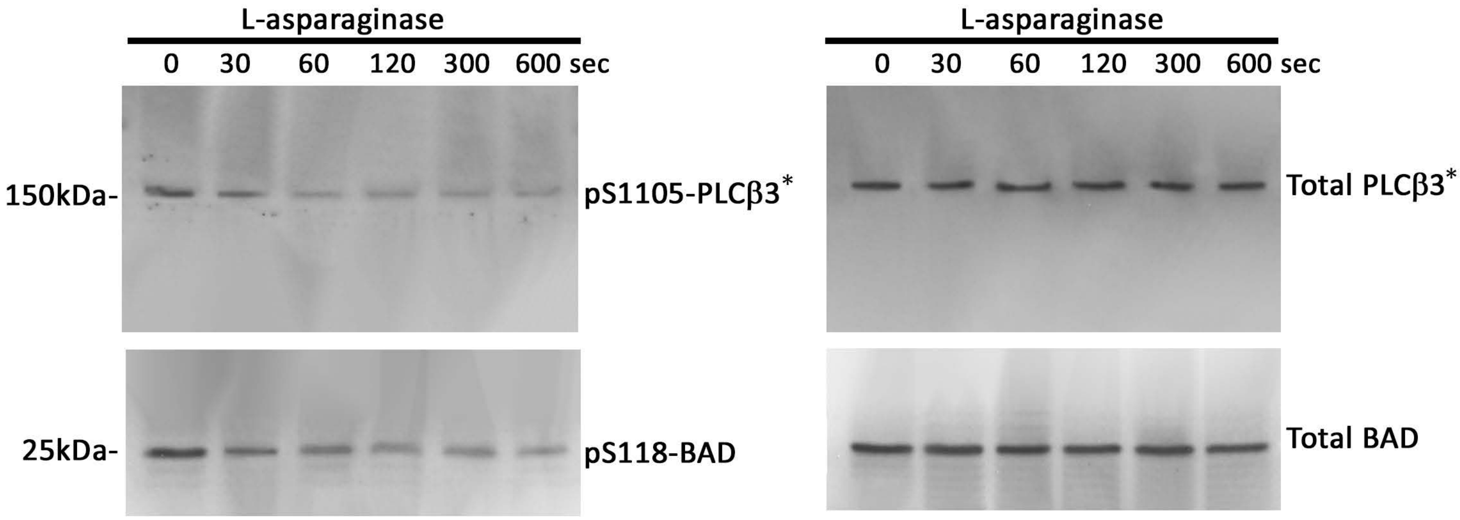

*some of the blots were cut to allow immunoblotting with different antibodies without reblotting the whole membrane

Figure 8B

*some of the blots were cut to allow immunoblotting with different antibodies without reblotting the whole membrane

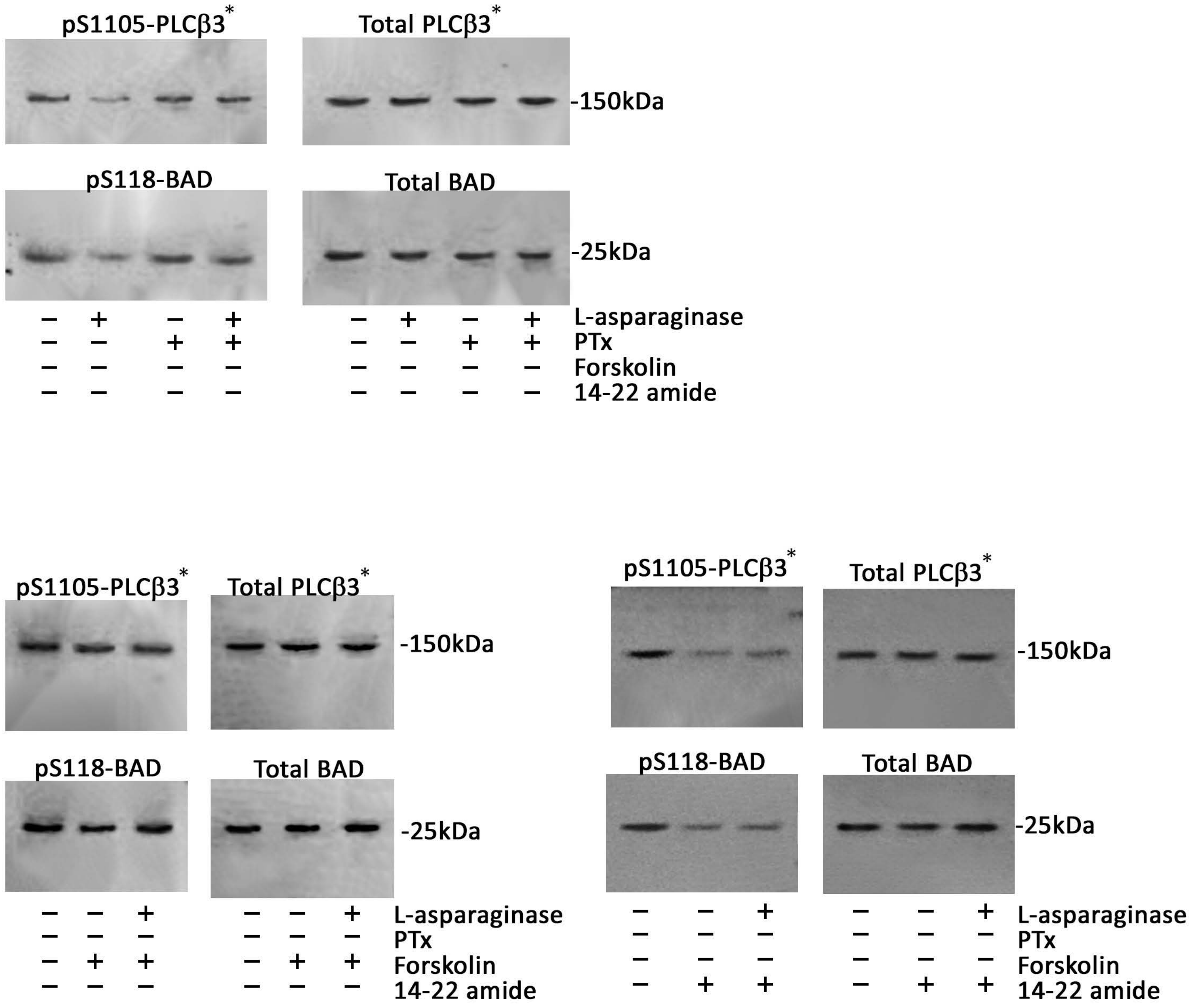

Supplementary Figure 1.

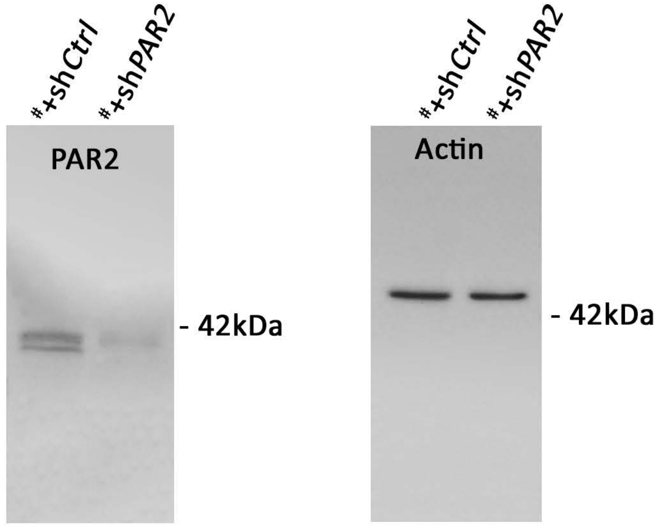

Supplementary Figure 6A

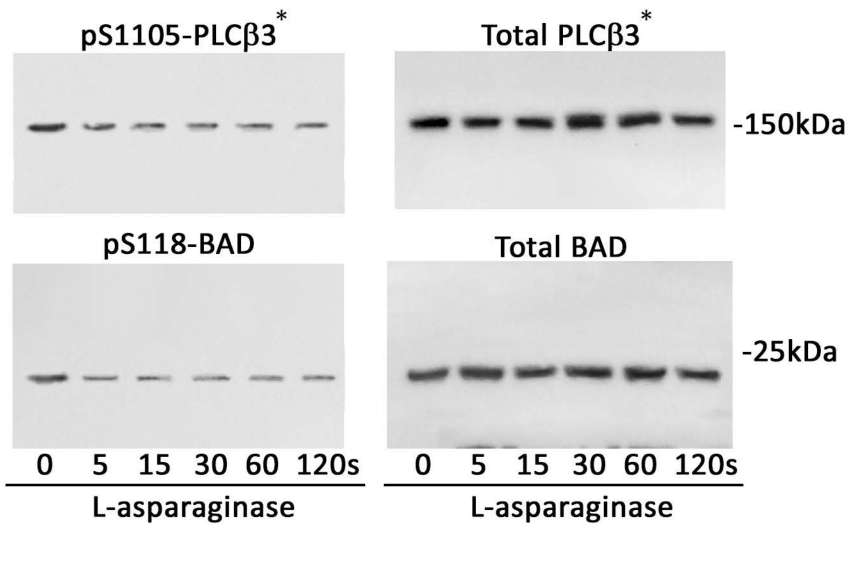

*some of the blots were cut to allow immunoblotting with different antibodies without reblotting the whole membrane

Supplementary Figure 6B

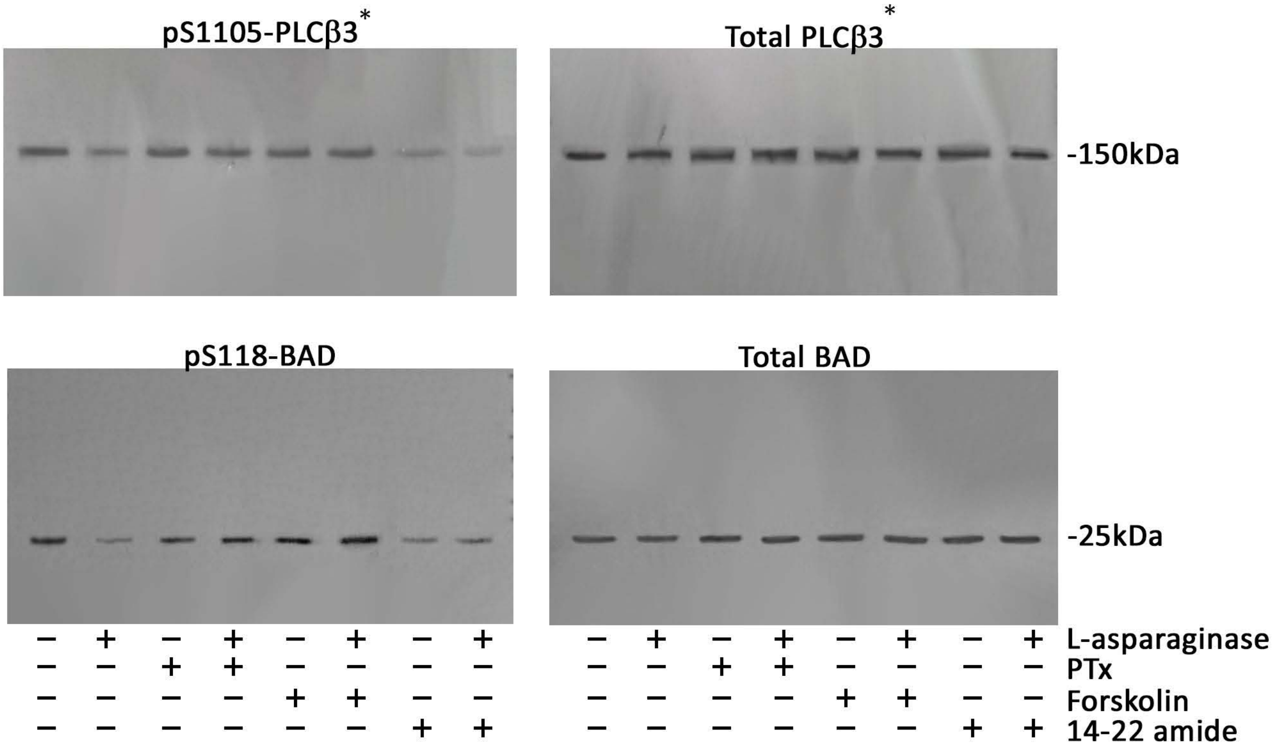

***some of the blots were cut to allow immunoblotting with different antibodies without reblotting the whole membrane.**
